# Supplementary material for: Regional Diversity in the Postsynaptic Proteome of the Mouse Brain
Source: Proteomes. 2018 Aug 1;6(3):31. doi: 10.3390/proteomes6030031 (PMC6161190; doi:10.3390/proteomes6030031)
Supplement: Supplementary file 1 [file proteomes-06-00031-s001.zip › SUPPL/20180630_Mouse Regions_SUPPLEMENTARY.pdf]

Supplementary Figures and Legends

Supplementary Figure S1.

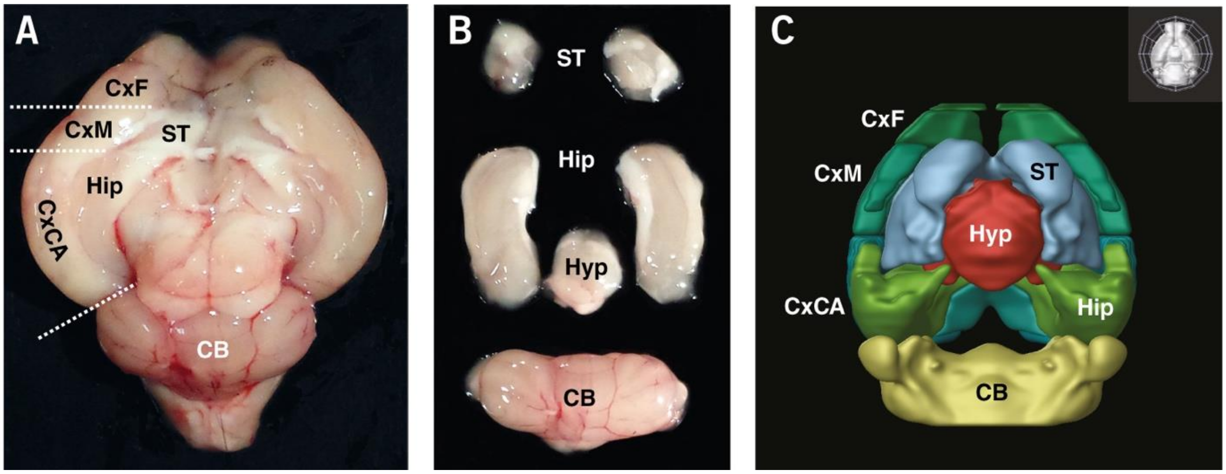

The seven regions dissected from the mouse brain. (A) Dorsal view of the unfixed brain after opening of the cortical halves from the midline to visualize the hippocampus (Hip) and striatum (ST) and three regions of the neocortex delineated by dotted white lines: frontal (CxF), medial (CxM) and caudal (CxCA) cortex. (B) Some of the regions collected, including the left and right striatum (ST), left and right hippocampus (Hip), hypothalamus (Hyp) and cerebellum (CB). (C) A 3D drawing of the horizontal-ventral to dorsal view of the mouse brain from the Allen Mouse Brain Atlas. The seven integral regions of the mouse brain are shown: CxF, CxM and CxCA (dark green), Hip (light green), ST (blue), Hyp (red) and CB (yellow).

Supplementary Figure S2.

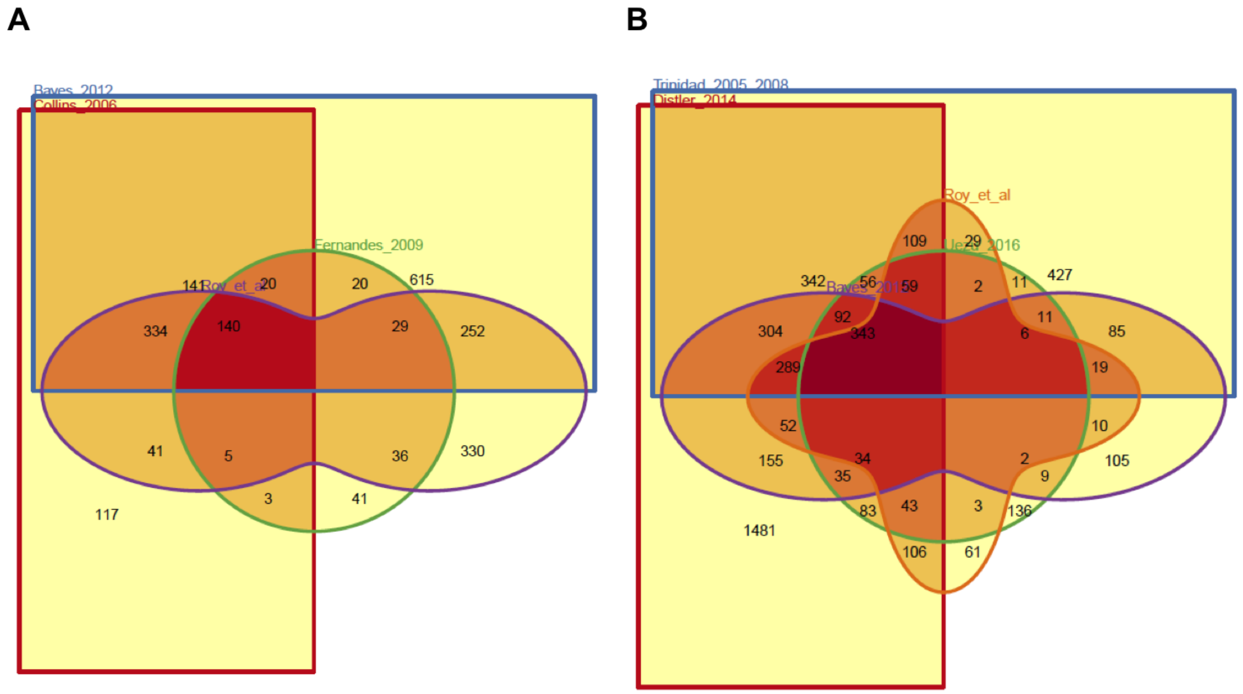

The overlap between proteomes obtained in this study and previous studies. (A) Overlap with studies previously performed on mouse PSD proteome by the same group (Collins et al., 2006, Fernandez et al., 2009, Bayes et al., 2012). (B) Overlap with studies of other groups performed on mouse PSD proteome (Trinidad et al., 2005, Trinidad et al., 2008, Bayes et al., 2012, Distler et al., 2014, Uezu et al., 2016).

Supplementary Figure S3.

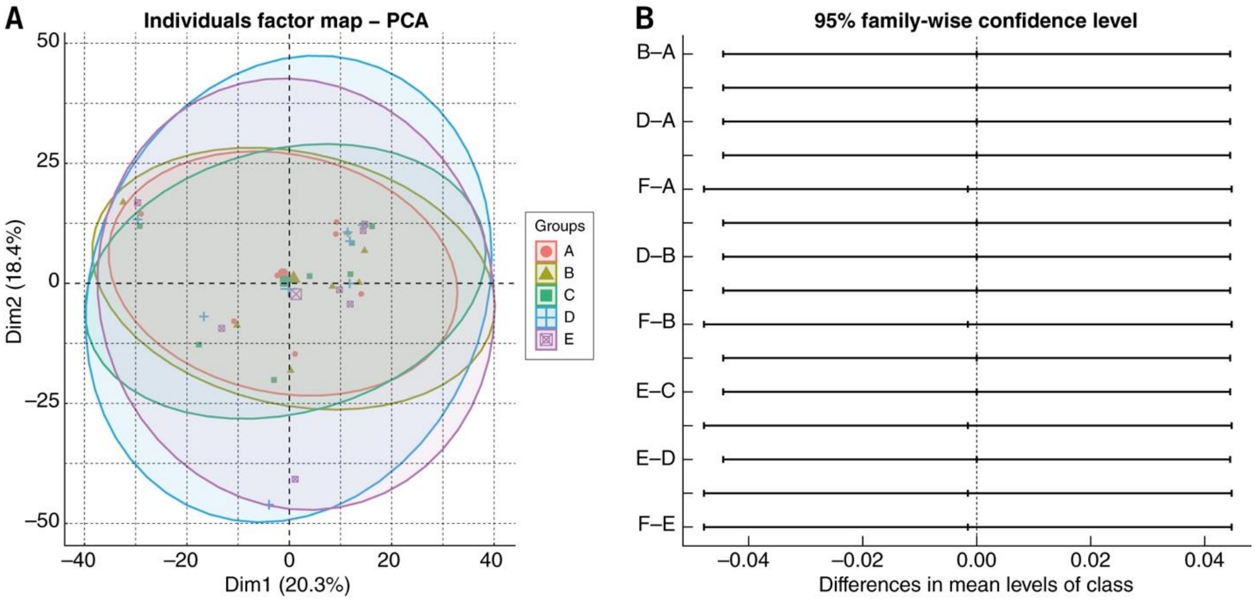

Principal component analysis (PCA) of the abundances of the 1,173 synaptic proteins quantified across the seven integral regions for five mouse brains (A-E). Brain F was not included in the PCA analysis as it was missing ST values. Confidence intervals for the mean differences as shown in panel B indicate that the major differences between groups are due to the position of outliers in the negative area.

Supplementary Figure S4.

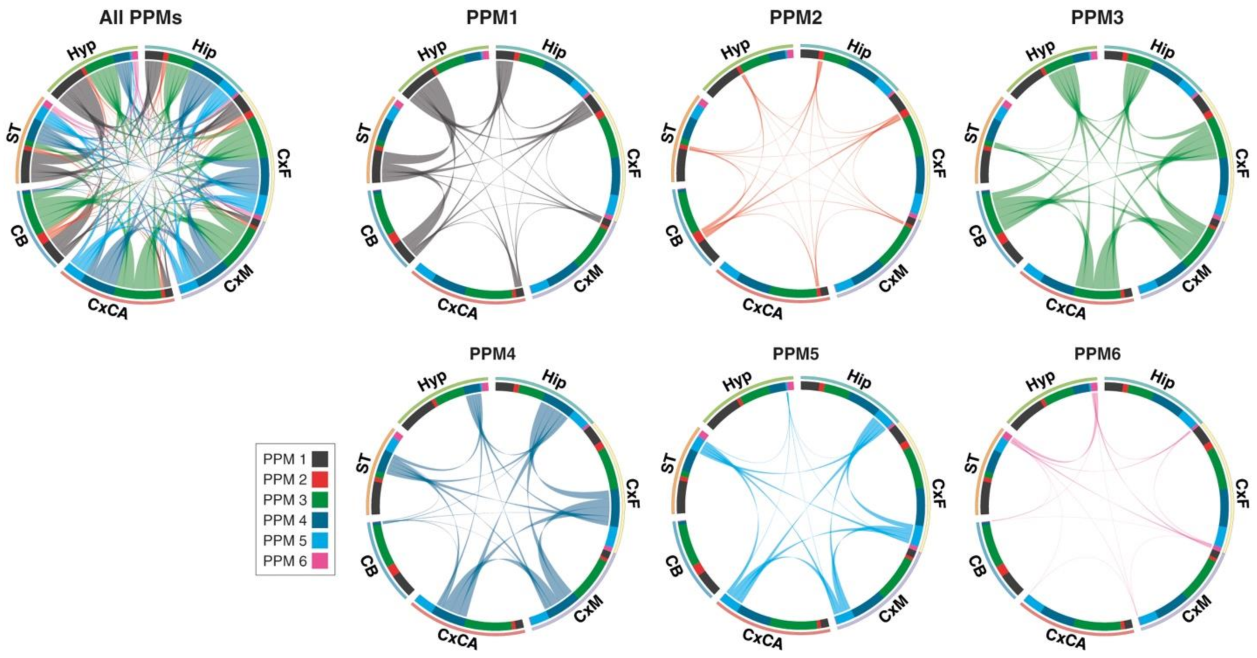

Circos plots showing differential abundance of the six postsynaptic proteome modules (PPMs) across mouse brain regions. The “all PPMs” circle (left) represents the overlap of individual PPMs, which are the plots shown on the right. Regions: frontal (CxF), medial (CxM) and caudal (CxCA) cortex, hippocampus (Hip), striatum (ST), hypothalamus (Hyp), cerebellum (CB). Color code of PPMs as in Figure 1B,C. Width of the link is proportional to the fraction of the regional proteins that contributed, while its color corresponds to the respective PPM.

Supplementary Table Legends

Supplementary Table S1. LC-MS/MS quantitation and comparison of the mouse synaptic proteome across the seven integral regions of the mouse brain by one-way ANOVA.

File: Supplemental Table 1_Mouse Brain Regions One Way ANOVA.xls

Data identifier: Source Data_Figure 1A and Sup Tables S3 and S4

Supplementary Table S2. New PSD proteins detected in this study compared to all other published mouse PSD studies. List of PSD proteins detected by LC-MS/MS that are novel to this study.

File: Supplementary Table 2_New PSD proteins detected in this study.xls

Data identifier: Supplementary Figure S2.

Supplementary Table S3. Differential stability analysis and functional enrichment of synaptic proteins. S3(A) Differential stability analysis of 1,173 synaptic proteins across the seven integral regions of the mouse brain. S3(B) Summary of the 572 synaptic proteins found to correlate (using Pearson's correlation coefficient (rho)) (Corr ≥0.5) across the seven integral regions of all six individual brains. S3(C) Functional enrichment in synaptic proteins that correlate between individual brains. S3(D) Summary of the 601 synaptic proteins that do not correlate (Corr ≤0.5) across the seven integral regions of all six individual brains. S3(E) Functional enrichment in synaptic proteins that do not correlate between individual brains.

File: Supplemental Table 3_Differential Stability Analysis.xls

Supplementary Table S4. Summary of the 868 synaptic proteins found to be differentially abundant across the seven integral regions of the mouse brain.

File: Supplemental Table 4_Summary of the 868 Differentially Abundant Synaptic Proteins by Brain Region

Supplementary Table S5. Module gene list and abundances for all six modules. Displayed are protein module gene lists and zero-centered abundances for 1,173 synaptic proteins quantified across the seven regions of the mouse brain.
File: Supplemental Table 5_Module gene list and abundances for all 6 modules.xls
Data identifier: Source Data Fig 2

Supplementary Table S6. Correlation of ABI functional connectivity with synaptic proteome abundances measured in this study.
File: Supplemental Table 6_Correlation of protein abundances with ABI data.xls
Data identifier: Source Data Fig 3

Supplementary Table S7. Functional Enrichment of ABI and Roy Correlates by Brain region.
File: Supplementary Table 7. Functional Enrichment of ABI and Roy Correlates by Brain region.xls
Data identifier: Source Data Fig 4

Supplementary Table S8. PSD protein interaction network analysis across the mouse brain.
File: Supplemental Table 8_Protein Interaction Network Analysis Across the Mouse Brain.xls
Data identifier: Source Data Supp Fig 5

Supplementary Table S9. Disease associated enrichment in the interactomes of the mouse PSD.
File: Supplemental Table 9_Disease associated enrichment in the interactomes of the mouse PSD.xls
Data identifier: Source Data Supp Fig 5

Supplementary Table S10. The “stable network”: postsynaptic proteome protein-protein interaction networks found to be enriched in at least four different regions of the mouse brain.

File: Supplemental Table 10_Composition of stable network.xls

Data identifier: Source Data Supp Fig 5
